# Effectiveness of influenza vaccine against laboratory-confirmed influenza, in the late 2011–2012 season in Spain, among population targeted for vaccination

**DOI:** 10.1186/1471-2334-13-441

**Published:** 2013-09-22

**Authors:** Silvia Jiménez-Jorge, Salvador de Mateo, Concha Delgado-Sanz, Francisco Pozo, Inmaculada Casas, Manuel Garcia-Cenoz, Jesús Castilla, Esteban Pérez, Virtudes Gallardo, Carolina Rodriguez, Tomás Vega, Carmen Quiñones, Eva Martínez, Juana María Vanrell, Jaume Giménez, Daniel Castrillejo, María del Carmen Serrano, Julián Mauro Ramos, Amparo Larrauri

**Affiliations:** 1 National Centre of Epidemiology, Institute of Health Carlos III, c/Monforte de Lemos no. 5, Madrid 28029, Spain; 2 Ciber Epidemiología y Salud Pública (CIBERESP), Ministry of Science and Innovation, Institute of Health Carlos III, Madrid, Spain; 3 National Centre for Microbiology, National Influenza Reference Laboratory, WHO-National Influenza Centre, Institute of Health Carlos III, Majadahonda, Madrid 28220, Spain; 4 Instituto de Salud Pública de Navarra, Navarra, Spain; 5 Servicio de Epidemiología y Salud Laboral. Secretaría General de Salud Pública y Participación. Consejería de Salud de Andalucía, Consejería, Spain; 6 Dirección General de Salud Pública, Consejería de Sanidad de Castilla y León, Spain; 7 Servicio de Epidemiología, Subdirección de Salud Pública de La Rioja, La Rioja, Spain; 8 Servicio de Epidemiología, Dirección General de Salut Pública, Baleares, Spain; 9 Servicio de Epidemiología. Dirección General de Sanidad y Consumo, Consejería de Bienestar Social y Sanidad, Ciudad Autónoma de Melilla, Spain; 10 Dirección General de Salud Pública, Servicio Extremeño de Salud, Junta de Extremadura, Spain

**Keywords:** Influenza, Vaccine effectiveness, Case–control studies, Sentinel networks, Discordant strain, Waning immunity

## Abstract

**Background:**

In Spain, the influenza vaccine effectiveness (VE) was estimated in the last three seasons using the observational study cycEVA conducted in the frame of the existing Spanish Influenza Sentinel Surveillance System. The objective of the study was to estimate influenza vaccine effectiveness (VE) against medically attended, laboratory-confirmed influenza-like illness (ILI) among the target groups for vaccination in Spain in the 2011–2012 season. We also studied influenza VE in the early (weeks 52/2011-7/2012) and late (weeks 8-14/2012) phases of the epidemic and according to time since vaccination.

**Methods:**

Medically attended patients with ILI were systematically swabbed to collect information on exposure, laboratory outcome and confounding factors. Patients belonging to target groups for vaccination and who were swabbed <8 days after symptom onset were included. Cases tested positive for influenza and controls tested negative for any influenza virus. To examine the effect of a late season, analyses were performed according to the phase of the season and according to the time between vaccination and symptoms onset.

**Results:**

The overall adjusted influenza VE against A(H3N2) was 45% (95% CI, 0–69). The estimated influenza VE was 52% (95% CI, -3 to 78), 40% (95% CI, -40 to 74) and 22% (95% CI, -135 to 74) at 3.5 months, 3.5-4 months, and >4 months, respectively, since vaccination. A decrease in VE with time since vaccination was only observed in individuals aged ≥ 65 years. Regarding the phase of the season, decreasing point estimates were only observed in the early phase, whereas very low or null estimates were obtained in the late phase for the shortest time interval.

**Conclusions:**

The 2011–2012 influenza vaccine showed a low-to-moderate protective effect against medically attended, laboratory-confirmed influenza in the target groups for vaccination, in a late season and with a limited match between the vaccine and circulating strains. The suggested decrease in influenza VE with time since vaccination was mostly observed in the elderly population. The decreasing protective effect of the vaccine in the late part of the season could be related to waning vaccine protection because no viral changes were identified throughout the season.

## Background

The viral antigens included in seasonal influenza vaccines are revised annually in anticipation of expected changes in circulating influenza viruses. Vaccine effectiveness (VE) cannot be presumed from historical data [1].

Evidence from trials and observational studies suggests that presently available influenza vaccines can provide only moderate overall protection against infection and illness [2]. However, influenza vaccination remains the most cost-effective public health prevention measure currently available for reducing the morbidity and mortality associated with influenza infection, as vaccination is strongly recommended every year by the international health authorities [3].

Influenza vaccination in Spain is annually offered free of charge to individuals at high-risk of influenza complications and to those over 59/ 64 years of age (depending on the region) [4].

Since the 2008–2009 season, Spain has been participating in the European Centre for Disease Prevention and Control(ECDC)-funded project I-MOVE [“Monitoring the influenza vaccine effectiveness in the European Union and European Economic Area (EU/EEA)”] with the cycEVA study within the framework of the Spanish Influenza Sentinel Surveillance System (SISSS) [5-9]. For at second year, in February 2012, the I-MOVE multicentre case–control study and cycEVA study were able to provide an intra-seasonal influenza VE estimate [10,11]. The results suggested a low-to-moderate protective effect of the seasonal 2011–2012 vaccine in preventing medically attended, laboratory-confirmed influenza in the target groups for vaccination. Final estimates suggested lower values later in the season [12-14]. The duration of the protection provided by influenza vaccines is debated and could be related to several factors, including age and the type/subtype of influenza infection [15].

We aimed to present the overall and age-specific end-of-season effects of the 2011–2012 influenza vaccine on preventing medically attended, laboratory-confirmed influenza-like illness (ILI) in the groups targeted for vaccination. Because the influenza season peaked unusually late in Spain, we also studied influenza VE in the early and late phases of the season and according to time since vaccination.

## Methods

### Study design and data collection

Seven regional influenza surveillance networks within primary care in different parts of Spain participated in the cycEVA study (test-negative design). The participating sentinel general practitioners and paediatricians (GPs) (231) adhered to a common protocol specifically designed for the European multicentre case–control study [16]. The physicians selected patients according to a definition based on the EU ILI case definition [7,12] and systematically swabbed the first two patients per week aged <65 years and all patients ≥65 years who presented to the GP’s office with ILI. Practitioners also collected the following information for each recruited patient: demographic and clinical data, vaccination status for 2011–2012 trivalent seasonal influenza vaccine (date of vaccination and type of vaccine received), laboratory data and data on potentially important confounders (previous influenza vaccination, the presence of any chronic condition, smoking history, any hospitalisation for chronic conditions in the previous 12 months, and the number of outpatient visits for any reason in the previous 12 months [7]. Missing information was not observed for the 2011–2012 vaccination status, and only two records were missing the previous vaccination status. The prevalence of missing data ranged from 0.7% to 1.8% for other variables related to possible confounding factors.

This study started in week 52/2011 (December 25, 2011), during which the ILI rate exceeded the winter baseline level and finished on week 18/2012 (May 6, 2012), which proceeded two consecutive weeks without any ILI cases testing positive for influenza.

### Identification of cases and controls

Cases were ILI patients who tested positive for influenza virus using reverse transcription polymerase chain reaction (RT-PCR) and/or cell culture. Controls were ILI patients who tested negative for any type of influenza virus. We considered a patient vaccinated if the patient had received the 2011–2012 influenza vaccine at least 14 days before ILI symptom onset.

### VE analysis

We restricted the analysis to patients who belonged to the target groups for vaccination and who were swabbed less than eight days after the onset of symptoms. We undertook two analyses of VE: for influenza (all strains) and for A(H3N2) influenza virus restricted to the weeks in which this strain predominated.

To check the effect of the late season on the effectiveness of the vaccine, we estimated influenza VE according to the 2011–2012 influenza season phase, splitting the season into two periods: an early phase before the epidemic peak (weeks 52/2011-7/2012) and a late phase after the peak (weeks 8-14/2012). To characterise a possible waning vaccine protection after vaccination, we also calculated influenza VE according to time since vaccination (the number of days between the date of vaccination and onset of symptoms) using three equal time intervals according to the distribution of the time since vaccination variable to facilitate comparisons between sub-groups. For trend evaluation we treated time since vaccination as a continuous variable in a logistic model to test the null hypothesis of beta = 0. We compared the characteristics of cases and controls using a t-test, Fisher’s exact test, a chi-squared test or the Mann–Whitney test, as appropriate. The age effect was taken into account by adjustment and by performing a stratified analysis of individuals <65 years and ≥65 years.

We estimated influenza VE as 1 minus the odds ratio (OR). To estimate the adjusted influenza VE, we used a logistic regression model that includes potential confounders that changed the crude OR by more than 10% and were related to both the exposure and the outcome [17]. We also adjusted for calendar time by using the week of swabbing as a categorical variable. Effect modification by age was assessed by likelihood ratio tests. We conducted all statistical analyses using Stata version 11.

### Laboratory methods

A subset of influenza isolates was sent to the National Influenza Centre (WHO Influenza Centre) for influenza-specific gene sequencing and genetic characterisation. The isolates were genetically characterised by sequencing the HA1 fragment of the viral haemagglutinin gene. Phylogenetic analysis was performed to characterise the specific strains of influenza A and B viruses.

We analysed the temporal trend of genetically characterised influenza A(H3N2) viruses in Spain in the entire SISSS using a simple linear regression model in which the logarithms of the relative weekly frequencies of the influenza A(H3N2) viruses, which were distinct from the vaccine virus, were considered as the dependent variable, and time (weeks) was the independent variable.

This observational study was part of Spanish influenza surveillance activities. Only anonymous personal information was collected and patients gave verbal informed consent to participate in the study. Consequently, no ethical approval by the Human Research Ethics Committee therefore was required.

## Results

### Description of 2011–2012 influenza season in Spain

**Figure 1:**
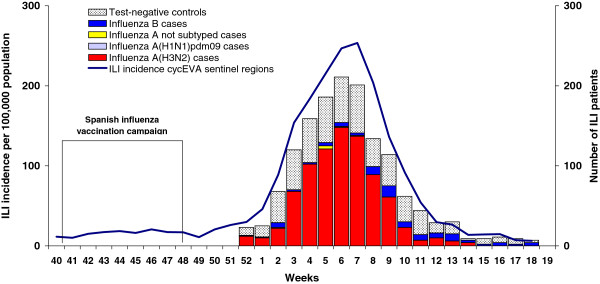
Controls and confirmed cases by type/subtype of influenza virus, and weekly incidence, cycEVA 2011-12 study, Spain.

In Spain, influenza activity in 2011–2012 reached its peak in mid-February (week 7/2012), with 251 ILI cases/100,000 people. The influenza season was largely dominated by influenza A(H3N2) with an increasing contribution of influenza B virus, which became dominant after the epidemic period. In the seven cycEVA regions we observed a similar viral circulation pattern and evolution of influenza activity (Figure 1).

### Patient characteristics

**Figure 2:**
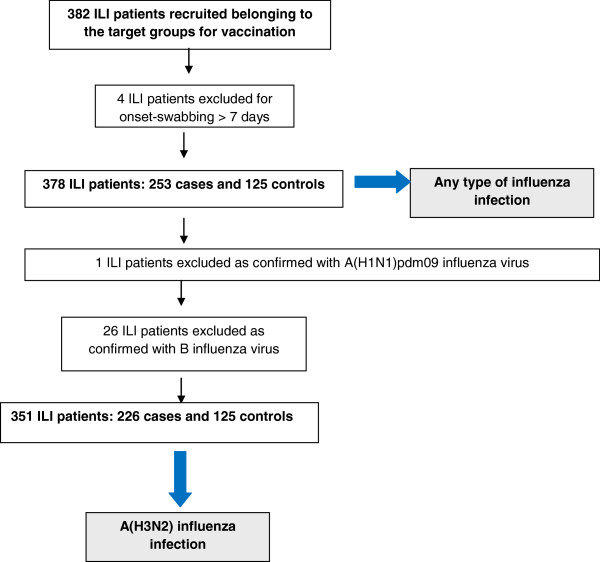
Flowchart of data exclusion and analysis outcomes, target groups for vaccination, cycEVA 2011-12 study, Spain.

In total, 231 practitioners agreed to participate in the study and 197 (85%) recruited at least one ILI patient. From the target groups for vaccination, 382 (27% of all patients) were recruited. All patients had information on their laboratory results and vaccination status, and only four individuals were swabbed more than seven days after symptom onset (Figure 2). After applying the exclusion criteria, we included 99% (378) of the recruited patients belonging to the target groups for vaccination in the analysis: 253 laboratory-confirmed influenza cases [226 A(H3N2), 1 A(H1N1)pdm09, and 26 B] and 125 test-negative controls.

The demographic and clinical characteristics of the 226 laboratory-confirmed influenza A(H3N2) cases and the 116 test-negative controls belonging to the population targeted for vaccination and included into the analysis, are displayed in Table 1. Controls were significantly younger than cases, with median ages of 53 and 63 years, respectively (P = 0.011). Compared with cases, controls were more likely to have certain chronic conditions (62.9% vs 51.8%; P = 0.049) and included a higher proportion of smokers (19.1% vs 11.2%; P = 0.046). Although the median time since vaccination was longer in cases than in controls (116 and 109 days, respectively), this difference was not statistically significant.

**Table 1:** Characteristics of influenza A(H3N2) confirmed cases (N = 226) and test-negative controls (N = 116) in target group for vaccination, cycEVA 2011–12 study (weeks 52/2011 – 14/2012), Spain

**Variables**	**Controls no./total no. (%)**^**a**^	**A(H3N2) cases no./total no. (%)**^**a**^	**P value**^**b**^
**Median age (range years)**	53 (3–87)	63 (3–93)	**0.011**
**Age group (years)**			
0–4	4/116 (3.2)	5/226 (2.2)	0.088
5–14	8/116 (6.5)	14/226 (6.2)	
15–64	69/116 (58.5)	107/226 (47.3)	
≥65	35/116 (31.8)	100/226 (44.2)	
**Sex: male**	52/116 (46.9)	106/226 (46.9)	0.716
**Any chronic condition reported**	73/116 (62.9)	117/226 (51.8)	**0.049**
**Pregnancy**	3/116 (2.6)	6/226 (2.6)	0.970
**Obesity**^**c**^	6/116 (4.9)	12/226 (5.3)	0.957
**Any hospitalization**	4/116 (3.4)	11/226 (4.9)	0.544
**Median GP visits (range number)**	5 (0–32)	5 (0–32)	0.782
**Smoker**	23/115 (19.1)	25/223 (11.2)	**0.046**
**Swabbing less 4 days**	108/116 (93.1)	217/226 (96.0)	0.240
**Median time since vaccination (range days)**	109 (39–151)	116 (62–166)	0.432
**Vaccine coverage**			
By seasonal vaccine			
Seasonal 2011-12	46/116 (39.7)	88/226 (38.9)	0.898
Seasonal 2010-11	40/116 (34.5)	75/224 (33.5)	0.853
By age groups			
0–4 years	1/4 (25.0)	2/5 (40.0)	0.635
5–14 years	1/8 (12.5)	2/14 (14.3)	0.907
15–64 years	19/69 (27.6)	19/107 (17.8)	0.124
≥65 years	25/35 (71.4)	65/100 (65.0)	0.487

GP: general practitioners and paediatricians.

^a^Cases and controls recruited between weeks 52/2011 – 14/2012 and with an interval between symptom onset and swabbing of less than eight days.

^b^Non parametric test of the median or Chi-square test or Fisher’s exact test, when appropriate.

^c^Defined as body mass index ≥ 40 kg/m^2^.

### VE results

The overall crude influenza VE among the target groups for vaccination against any type or A(H3N2) influenza virus was estimated at 8% (95% CI, -43 to 41) and 3% (95% CI, -53 to 39), respectively. The adjusted influenza VE estimates adjusted for age group, smoking habit and week of swabbing were 47% (95% CI, 7–70) against any type of influenza virus and 45% (95% CI, 0–69) against A(H3N2) influenza virus (data not shown).

Adjusted influenza VE estimates against A(H3N2) according to time since vaccination were 52% (95% CI, -3 to 78), 40% (95% CI, -40 to 74) and 22% (95% CI, -135 to 74) for 3.5 months, 3.5-4 months, and >4 months since vaccination, respectively (data not shown) (p for trend = 0.109).

The analysis of influenza VE according to time since vaccination and age group is shown in Table 2. Among individuals ≥65 years the adjusted VE against A(H3N2) decreased from 85% (95% CI, 18–97), for patients vaccinated three months before the onset of symptoms to a null estimate for individuals vaccinated more than four months before the onset symptoms (p for trend = 0.211). Decreasing VE with time since vaccination was not observed in patients <65 years (p for trend = 0.335) (Table 2). Regarding effect modification, there was no evidence that VE varied by age (p = 0.80).

**Table 2:** Effectiveness of the trivalent influenza 2011–12 vaccine against influenza A(H3N2) in target group for vaccination by age group, according to the time since vaccination, cycEVA study (weeks 52/2011 – 14/2012), Spain

**Study population**	**Time since vaccination**	**Cases and controls (N/N)**	**Vaccinated cases and controls (N/N)**	**OR**	**Influenza VE % (95% CI)**	**Trend p value**^**c**^
≥65 years	**49–88 days**	39/17	4/7	Crude	84 (33;96)	0.211
				Adjusted	85 (18;97)^a^	
	**89–127 days**	72/24	37/14	Crude	24 (−92;70)	
				Adjusted	33 (−102;78)^a^	
	**128–166 days**	58/14	23/4	Crude	−64 (−487;54)	
				Adjusted	−376 (−4332;49)^a^	
<65 years	**39–75 days**	104/64	1/4	Crude	85 (−33;98)	0.335
				Adjusted	84 (−138;99)^b^	
	**76–111 days**	114/66	11/6	Crude	−7 (−203;62)	
				Adjusted	19 (−149;73)^b^	
	**112–148 days**	113/71	10/11	Crude	47 (−32;79)	
				Adjusted	58 (−19;86)^b^	

^a^Model adjusted for age groups (65–75, 76–85, 86–95 and 95–105 years), and week of swabbing. ^b^Model adjusted for age groups (0–4, 5–14, 15–44 and 45–64 years), and week of swabbing. ^c^Test for trend using time since vaccination as continuous.

In the early influenza phase the adjusted influenza VE against A(H3N2) was 52% (95% CI, 4–76), compared with 28% (95% CI, -124 to 77) in the late phase (data not shown).

Analysis according to time since vaccination in the different influenza phases showed that in the early influenza phase, adjusted VE estimates against A(H3N2) decreased with time since vaccination from 95% (95% CI, 45–99) at three months since vaccination to 36% (95% CI, -71 to 76) at more than 3.5 months since vaccination (Table 3). This decreasing trend with time since vaccination was not statistically significant (p for trend = 0.119). In the late influenza phase, adjusted influenza VE estimates were very low or null in the three studied strata (Table 3).

**Table 3:** Effectiveness of the trivalent influenza 2011–12 vaccine against influenza A(H3N2) in target group for vaccination by time since vaccination, in the early and late phase of the season, cycEVA study (weeks 52/2011 – 14/2012), Spain

**Influenza activity phase**^**a**^	**Time since vaccination**	**Cases and controls (N/N)**	**Vaccinated cases and controls (N/N)**	**OR**	**Influenza VE % (95% CI)**	**Trend p value**^**d**^
**Early phase**	**45–75 days**	110/63	1/8	Crude	94 (48;99)	0.119
				Adjusted	95 (45;99)^b^	
	**76–105 days**	128/66	19/11	Crude	13 (−96;53)	
				Adjusted	48 (−31;80)^c^	
	**106–135 days**	131/64	22/9	Crude	−23 (−186;47)	
				Adjusted	36 (−71;76)^c^	
**Late phase**	**39–81 days**	29/16	0/1	Crude	0	0.518
				Adjusted	0	
	**82–123 days**	47/21	18/6	Crude	−55 (−372;49)	
				Adjusted	−9 (−379;75)^b^	
	**124–166 days**	55/26	26/11	Crude	−22 (−213;52)	
				Adjusted	15 (−214;77)^b^	

^a^Early phase: December 2011 – first half of February 2012; Late phase: Second half of February – April 2012. ^b^Model adjusted for age groups (0–4, 5–14, 15–64 and >64 years), and week of swabbing. ^c^Model adjusted for age groups (0–4, 5–14, 15–64 and >64 years), smoking habit, and week of swabbing. ^d^Test for trend using time since vaccination as continuous.

### Laboratory results

**Figure 3:**
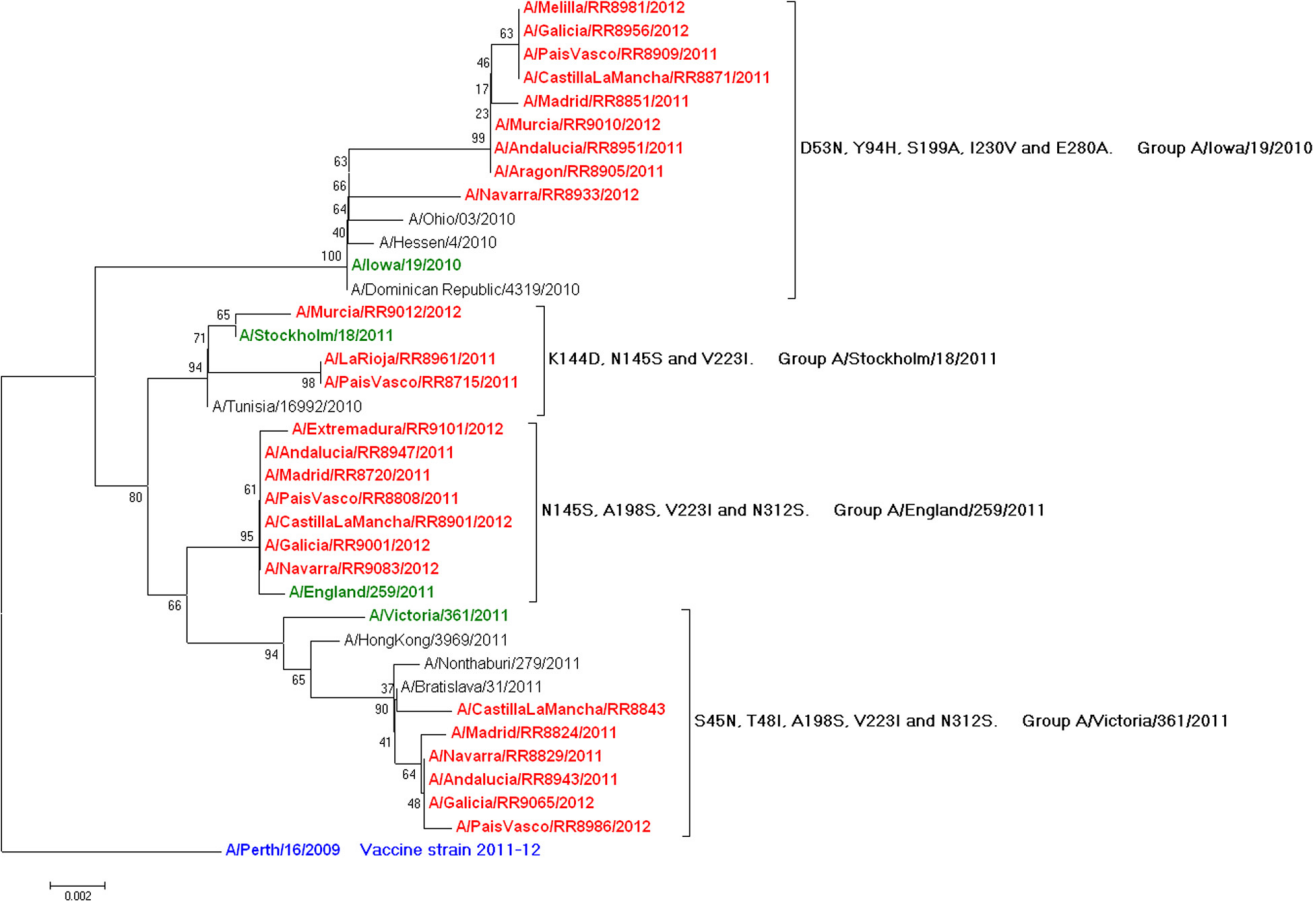
Phylogenetic tree of the influenza A(H3N2) viruses HA1 fragment of the hemagglutinin gene, cycEVA 2011-12 study, Spain.

Sequence analysis of the product of amplification targeting the full-length HA1 segment of hemagglutinin showed that most of the studied influenza A virus strains clustered into three genetic groups defined by specific amino acid mutations compared with A/Perth/16/2009 (Figure 3). Among the 127 A(H3N2) sequenced strains, 40% clustered into the group A/England/259/2011, 36% clustered into the group A/Victoria/361/2011 and 22% clustered into the group A/Iowa/19/2010. The remaining two A(H3N2) viruses (1%) clustered into the group A/Stockholm/18/2011.

The weekly proportion of influenza A(H3N2) viruses distinguishable from the vaccine virus in Spain was similar during the entire study period, ranging from 91%-100%, with a not significant weekly percentage change of −0.21% (95% CI, -0.64 to 0.22).

The only characterised A(H1N1)pdm09 virus clustered into A/StPetersburg/100/2011. Regarding influenza B, the Yamagata lineage (n = 16, 94%) viruses predominated over those of the Victoria lineage (n = 1, 6%). Most of the Yamagata lineage viruses clustered into the B/Bangladesh/3333/2007 genetic clade, and only one clustered into the B/Brisbane/3/2007 group.

No specific amino acid differences were observed in virus strains among age groups patients or between vaccinated and non vaccinated patients.

## Discussion

The results for the 2011–2012 season of the cycEVA study show a low-to-moderate protective effect for the 2011–2012 seasonal trivalent influenza vaccine against medically attended, laboratory-confirmed influenza in the target groups for vaccination, consistent with Spanish and European estimates [11-14]. In a late influenza season with a limited match between vaccine and circulating strains, we may suggest waning protection of the influenza vaccine 2011–2012 in the elderly with time since vaccination, although the trends were not statistically significant [12-14].

Although this study runs within the framework of the current SISSS, it is an observational study that followed a common protocol to be part of the European multicentre case–control study I-MOVE. Using the EU ILI case definition, GPs performed systematic sampling and collected high quality information on the main confounding factors described in the literature, thus reducing possible confounding bias.

By restricting the study to the epidemic period we also reduced the possible bias resulting from the inclusion of ILI patients when influenza viruses are not circulating [18]. During this period of intense influenza activity, influenza positivity was higher than 50%, such that a higher number of cases than controls was included in the analysis.

The test-negative study design is becoming an increasingly well-established approach to measuring influenza VE and generates the highest estimates of VE [18-22]. This design avoids confounding by the propensity to seek care within the control group, which is negative for influenza, thus providing better comparability with the confirmed cases [23-27]. In addition, study participants were selected by practitioners according to a systematic sampling procedure before either the patient or the physicians knew the case and control status of the patients. This protocol should minimise selection bias [23]. By restricting our analysis to ILI patients swabbed less than eight days after the onset of symptoms, we tried to minimise the possibility of misclassification because of false-negative RT-PCR results that could contribute to an underestimation of influenza VE. In addition, adjusting for swabbing week helped to overcome another possible limitation of the test-negative design by controlling the analysed data for calendar time [18]. Nevertheless, as with any observational design, we cannot rule out residual bias and confounders [23,28].

The 2011–2012 influenza season was characterised by several noteworthy aspects in Spain. First, this season was notably late, as in most of the northern hemisphere’s temperate zone with the exception of North Africa [29]. The epidemic peak was not reached in Spain until mid-February 2012, whereas peaks are usually in late December or early January [30]. Second, there was a predominant circulation of A(H3N2) influenza virus with a minimal contribution of the A(H1N1)pdm09 influenza subtype, which has been the predominant subtype since the 2009 pandemic. Third, there was a limited match between the vaccine and circulating A(H3N2) strains [29,31].

Our global adjusted influenza VE estimate against A(H3N2) influenza infection, 45% (95% CI, 0–69), was consistent with the VE estimated in Australia for the 2011 season against influenza A(H3) (58%, 95% CI, -53 to 89) [32] and with the results of previous studies in years with a predominant circulation of seasonal influenza A(H3N2) virus, which determined influenza VE to range from 10% to 68%, depending on the degree of antigenic match [20,21,24,33]. Although the effectiveness of the influenza vaccine is often less pronounced during seasons with antigenic mismatch between the vaccine and the circulating strains [34], in certain influenza season’s, antigenic changes occur without resulting in any apparent loss of influenza VE [35].

Several factors might have contributed to the low to moderate protective effect of the 2011–2012 trivalent influenza vaccine. Firsly, the circulating A(H3N2) influenza viruses in Spain clustered into several genetic groups that were reported to be antigenically and genetically distinct from the vaccine virus A/Perth/16/2009 [31]. This limited match was observed globally in the northern hemisphere [31], resulting in a change in the WHO recommended A(H3) vaccine strain for 2012–2013 in the northern hemisphere [36].

Second, in an unusually late influenza season [29,30], with a time lag between the vaccination campaigns and the start of the epidemic that was longer than in previous seasons, our results suggested a decrease in the protective effect of the 2011–2012 trivalent influenza vaccine with time since vaccination. This pattern was also observed in other studies [12-14].

A decreasing influenza VE over time could be related to increasing changes in circulating viruses towards the end of the season and/or potentially waning immunity in the months following vaccination [12,29]. Phylogenetic analyses of the circulating influenza viruses in Spain did not support the hypothesis of an increased emergence of antigenically drifted A(H3) variants during the influenza season in Spain. There was a presence of mismatched influenza viruses since the beginning of the 2011–2012 Spanish influenza season, with a similar weekly proportion of circulating changed A(H3N2) influenza strains throughout the entire study period.

Re-analysing influenza VE data for the preceding season 2010–2011 [7] we also observed decreasing influenza VE against A(H1N1)pdm09 in target groups for vaccination, from 62% (95% CI, -1 to 85) to 44% (95% CI, -122 to 86) for those vaccinated within three months or more than three months before the onset of symptoms, respectively. This lower decrease in VE over time (26%) observed in the previous season relative to the VE in our study for the 2011–2012 season (46%, data not shown) highlights the possibility that even in a usually timed season with a predominant circulation of a well-matched A(H1N1)pdm09 influenza virus, the influenza vaccine exhibits a degree of waning immunity.

To further explore the decrease in the protective effect of the vaccine with time since vaccination, we determined influenza VE by the phase of the season. We observed a decreasing influenza VE with time since vaccination during the early phase, from a high influenza VE of 95% (95% CI :45–99) at three months since vaccination, to a lower influenza VE point estimate of 36% (95% CI: -71 to 76) at more than 3.5 months since vaccination.

In the late phase, influenza VE estimates were compatible with null vaccine protection since shortly after vaccination. This finding is consistent with the finding that patients included in the late-phase subgroup had a median time since vaccination that was nearly one month longer that that in individuals in the early phase (128 days, range: 39–166 days vs 103 days, range: 45–135 days, respectively).

Together, these findings could reinforce the hypothesis of the possibly waning protection of the influenza vaccine. However, these results must be interpreted with caution because the study was limited by its small sample size. Therefore, although point estimates showed a substantial decrease with time since vaccination in the early phase, we could not demonstrate a significant decreasing influenza VE trend over time.

By age group, a decline in influenza VE with time since vaccination was also observed only in patients ≥65 years, although interpretation is limited by small sample size, which likely precluded the observation of a significant trend with time since vaccination [37].

A significant reduction in antibody titres 5–6 months after vaccination and, therefore, waning immunity following seasonal vaccination has been demonstrated in the elderly [38,39].

However, in our study, we also obtained a higher influenza VE point estimate shortly after vaccination among the elderly compared with aged <65 years patients: 71% (95% CI: 6–91) and 45% (95% CI: -114 to 87), respectively [40]. Conflicting results have been reported concerning the association between older age and the response to influenza vaccines. Several authors have found a reduced response in aged subjects, but others have reported no difference or even better results compared with younger control subjects [15,41]. Other studies showed that subjects vaccinated in every epidemic season for several years were protected against influenza despite low titres of anti-haemagglutinin antibodies [42]. Recently, studies performed during 2010–2011 influenza season demonstrated a higher influenza VE in patients vaccinated with both the current 2010–2011 and the previous 2009–2010 influenza vaccines in all age groups [7,43] and in a population with major chronic conditions [44]. In our study, a higher proportion of the elderly population (59%) compared with individuals <65 years (15%) was vaccinated with both the 2011–2012 vaccine and previous 2010–2011 seasonal influenza vaccines (p = 0.000). This factor could explain the higher influenza VE estimate obtained in the elderly relative to the younger group.

It is worth noting that currently there are few studies analysing influenza VE according to the time when the vaccination was given [12-14]. In addition, although levels of antibodies to seasonal inactivated influenza vaccine decline in the months following vaccination, this phenomenon does not necessarily reflect clinical VE [15]. Consequently, although limited by their statistical power, our results contribute to the currently available scientific evidence on influenza VE, which have been poorly studied so far. Nevertheless, how antigenic drift in circulating influenza strains could affect influenza VE in a late season remains unclear. Further studies are needed to elucidate the impact of these and other possible factors on the protective effect of the influenza vaccine.

Our results were also limited by low vaccine coverage (VC), especially in individuals <65 years. In addition, we cannot extrapolate influenza VE estimates for older people to all elderly populations [5] because influenza VE in the ≥65-years-old test-negative controls was higher than VC in the same age group belonging to the GPs’ catchment area (70% vs. 56%). Another limitation of our study arises from the recommendation of swabbing to all ILI patients ≥65 years, a subgroup of the population targeted for vaccination that was included in our study, what could have introduced a selection bias that affected the influenza VE estimates. However, we believe that the target group for vaccination is a homogeneous study population with regard to vaccination, the main exposure of interest, because the study participants had more equal access to vaccination than the total population.

Because annual influenza vaccination is recommended by public health authorities, it is crucial to annually evaluate influenza immunisation programs and issue recommendations. This study has fulfilled this mission over the past four years by developing and implementing a sustainable system for annually assessing of influenza VE vaccination in Spain and Europe, as part of the I-MOVE project. Over two consecutive years, preliminary and end-of-season influenza VE estimates supported the feasibility of generating and disseminating preliminary influenza VE estimates while virus circulation is ongoing [11,45].

The low-to-moderate protective effect of the 2011–2012 influenza vaccine in Spain that was observed in this study is in line with evidence from trials and observational studies to date [2]. This finding highlights suboptimal vaccine performance in most years within the presently available influenza vaccines, with performance seldom exceeding 60%, thereby underscoring the urgent need for better and longer-lasting protective vaccines [22,46]. Moreover, results on influenza VE, together with virological studies, should contribute to decision-making for the annual selection of influenza vaccine strains.

After five editions, the test-negative design of the cycEVA study has provided reliable information on annual influenza VE in Spain and may have important implications for the design of control influenza strategies.

## Conclusions

In conclusion, the 2011–2012 trivalent influenza vaccine in Spain showed a low-to-moderate protective effect in the groups for whom vaccination was recommended.

Influenza VE estimates in the early phase of the season, for patients vaccinated within three months of the onset of symptoms reinforce the importance of official recommendations for annual influenza vaccination. Decreasing influenza VE over time in the elderly population has been suggested, but it is not possible to disentangle the respective roles of the waning protection of the influenza vaccine, changes in the circulating viruses during the season and other unknown factors.

Our findings have important implications that can guide national policy makers in the design of influenza control strategies when facing future late influenza seasons. Moreover, the data support an urgent need for the development of new influenza vaccines providing better and longer-lasting protection.

## Competing interests

The authors declare that they have no competing interests.

## Authors’ contributions

All the authors participated in the design, implementation and interpretation of the study. SJ-J wrote the first draft of the article and had full access to all the study data and take responsibility for the data and accuracy of the data analysis, as well as the interpretation of the results obtained revising all drafts of the article. AL had full access to all the study data and take responsibility for the data and accuracy of the data analysis, as well as the interpretation of the results obtained revising all drafts of the article. SM discussed analyses and interpreted the results. CD-S discussed analyses and interpreted the results. FP collected and interpreted virological data. IC collected and interpreted virological data. MGC collected and interpreted epidemiological data. JC collected and interpreted epidemiological data. EP collected and interpreted epidemiological data. VG collected and interpreted epidemiological data. CR collected and interpreted epidemiological data. TV collected and interpreted epidemiological data. CQ collected and interpreted epidemiological data. EM collected and interpreted epidemiological data. JMV collected and interpreted epidemiological data. JG collected and interpreted epidemiological data. DC collected and interpreted epidemiological data. MCS collected and interpreted epidemiological data. JMR collected and interpreted epidemiological data. All authors critically reviewed the manuscript and approved the final draft. SJ-J and AL are equally responsible for this article.

## Pre-publication history

The pre-publication history for this paper can be accessed here:

http://www.biomedcentral.com/1471-2334/13/441/prepub
